# An integrative analysis to reveal that CLEC2B and ferroptosis may bridge the gap between psoriatic arthritis and cancer development

**DOI:** 10.1038/s41598-022-19135-2

**Published:** 2022-08-27

**Authors:** Xiaobin Li, Xiaohua Tao, Xiaoxia Ding

**Affiliations:** 1 Department of Dermatology and Venereology, Zhejiang Provincial People’s Hospital, Affiliated People’s Hospital, Hangzhou Medical College, Hangzhou, 310000 Zhejiang China; 2 Department of Orthopedic Surgery, Linping Campus, The Second Affiliated Hospital of Zhejiang University School of Medicine, Hangzhou, 31000 Zhejiang China

**Keywords:** Computational biology and bioinformatics, Genetics, Diseases

## Abstract

Patients with cutaneous psoriasis (PsC) and psoriatic arthritis (PsA) are reported with increased cancer risk, but the underlying mechanism is less clear, especially the association between the presence of PsA and cancer risk. Motivated by the role of ferroptosis in the progression of cancers as well as inflammation response in psoriasis, this experiment attempts to investigate the relationship between ferroptosis regulators and hub genes in PsA by bioinformatic analysis. The findings revealed an exclusive correlation between CISD1 (ferroptosis regulator) and CLEC2B (hub gene) in PsA group as well as multiple cancer types. Furthermore, CLEC2B was discovered differentially expressed in a variety of cancers and is closely associated with immune cell infiltration as well as immune checkpoints. These results indicate that ferroptosis may act as a bridge between psoriatic arthritis and the onset of certain malignancies.

## Introduction

Psoriasis disease (PsD) is a polygenic, hyperproliferative chronic inflammatory disease with unknown etiology^1^, which affects 2–3% of the population. A number of comorbid conditions, such as depression, cardiovascular disease, and particularly cancer, compound the impact of PsD. The incidence of cancer and cancer-related mortality seem to be higher in people with PsD, but the underlying association is much less clear^2,3^. To improve the understanding of the underlying mechanisms of this increased risk. Cutaneous psoriasis (PsC) and psoriatic arthritis (PsA) are fall under the umbrella of psoriasis disease^4,5^. PsA is a chronic, progressive, inflammatory arthritis associated with PsC that can impact a variety of organ systems^6^. To date, little is known about the cancer-related genomics in patients with PsD, even less is clear whether presence of PsA is associated with cancer risk in psoriasis patients.

Ferroptosis, a new form of regulated cell death that is iron- and reactive oxygen species (ROS)-dependent, has attracted much attention in cancer development and treatment^7^. According to research, ferroptosis is likely to inhibit tumor formation and/or progression, which means inducing ferroptosis is a promising anticancer strategy. Activation of lipid ROS and iron metabolism or suppression of antioxidant metabolism is the primary approach of ferroptosis induction based cancer therapy^8,9^. Interestingly, ROS occurs systemically and locally in psoriasis^10^. Many current treatments further increase oxidative stress and ROS production to accelerate the removal of damaged epidermal cells^11^. The induction of ferroptosis in psoriatic keratinocytes, particularly lipid peroxidation, has been proven to contribute to inflammatory responses^12^. Therefore, we propose the hypothesis that association analysis between hub genes contributed to PsA development and ferroptosis regulators may provide a new perspective on the pathogenesis of high cancer risk in patients with PsA.

There is growing interest in microarray platforms as a way to detect genetic alterations and to determine biomarkers for many diseases^13^. Several biomarkers and pathways have been implicated in the development of PsD in previous studies on microarray data^14,15^. Moreover, genetic and proteomic studies also confirmed the overlap and differences between PsC and PsA. Specifically, whole blood gene expression analysis suggests that innate immunity may play an important role in the pathogenesis of PsA through TLR signaling, NF-kB, and various chromatin remodeling complexes. NOTCH2NL, HAT1, CXCL10, and SETD2 were found most notably associated with PsA patients^16^. But still no studies have been conducted on the differential analysis of ferroptosis regulators between PsA and PsC.

In this study, we set up 2 comparisons including PsA-CL (normal controls) group and PsA-PsC group. Overlapped differentially expressed genes obtained from aforementioned two comparisons were considered as important genes contributed to PsA development, which were used for further analysis^17–19^, including GSEA/DO enrichment analysis, and PPI network construction to obtain hub genes. Subsequently, association analysis of hub genes with differentially expressed ferroptosis regulators was performed. Co-expression interactions in pan-cancer were analyzed using genes with exclusive correlation in PsA to further narrow down the hub genes. CLEC2B, the final standout, was also discovered to be differently expressed in a variety of malignancies and to have a substantial link to prognosis and immune infiltration in a variety of tumors.

As a result, we propose that CLEC2B, via ferroptosis regulators, may be a bridge gene for disease progression and a high cancer risk in psoriatic arthritis.

## Materials and methods

**Figure 1:**
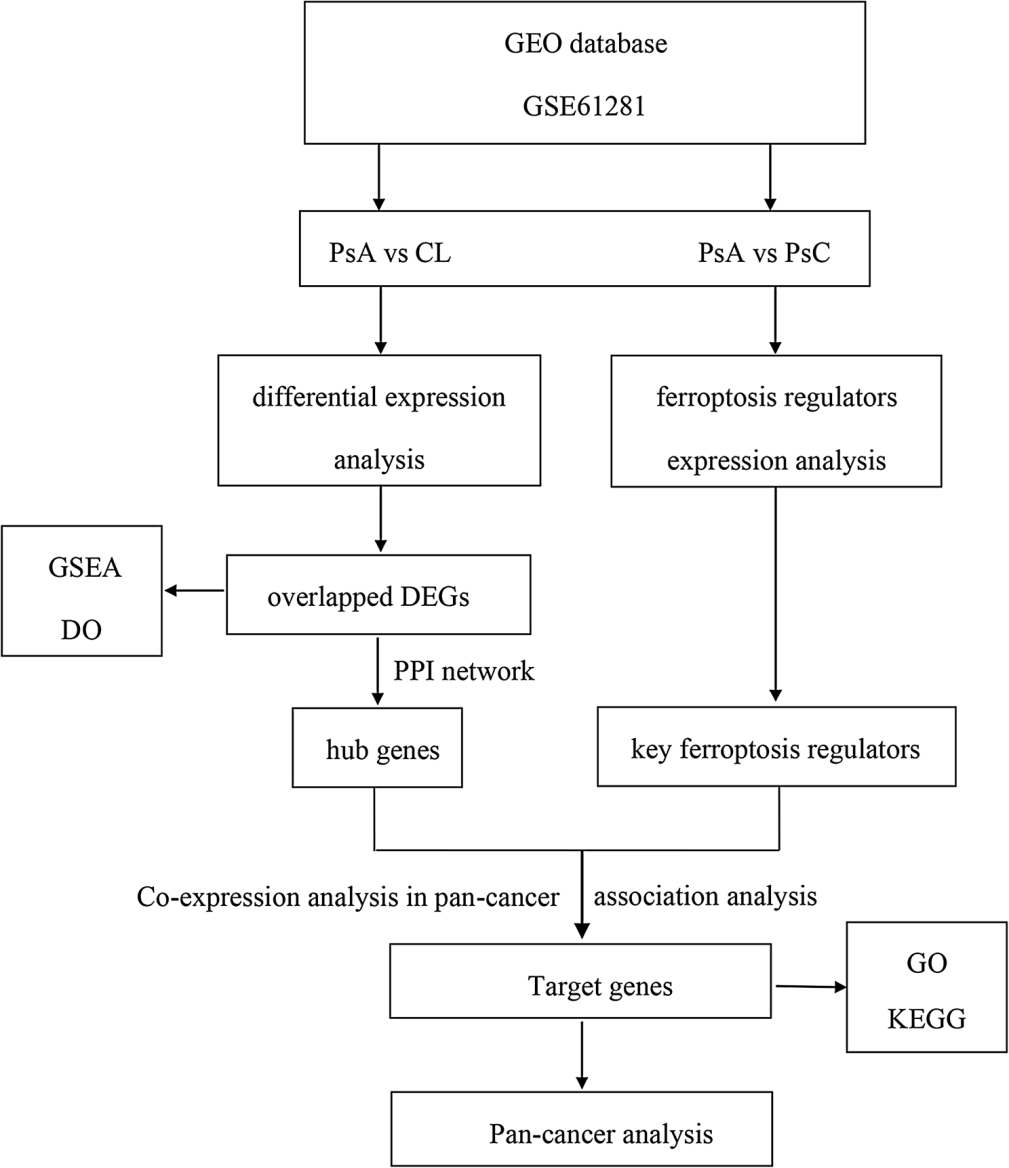
Flowchart of methods.

The flow chart of this study is shown in Fig. 1.

### Dataset collection

Gene expression datasets were collected from the Gene Expression Omnibus database (http://www.ncbi.nlm.nih.gov/geo)^20^. The uniformly normalized pan-cancer dataset was downloaded from the UCSC (https://xenabrowser.net/) database^21^: TCGA TARGET GTEx (PANCAN, N = 19,131, G = 60,499).

### Data processing and differential expression analysis

Raw data were processed and analyzed using R (version 4.0.2)^22^. Firstly, the RMA algorithm^23^ was used for background correction and data normalization and the result was show through box plot. Then, the “limma” R package^24^ was used to identify DEGs (|Log2FC| ≥ 1, P < 0.05) between each group in the dataset, and a volcano map and heatmap of DEGs was drawn respectively.

### Functional correlation analysis

Gene Ontology (GO) on DEGs were performed respectively using “clusterProfiler” package^25^. Kyoto Encyclopedia of Genes and Genomes (KEGG) pathway analyses were performed using GAGE^26^ followed by visualization with the ggplot2 R package^27^. Annotation of the genes and pathways was provided by the KEGG database (http://www.genome.jp/kegg)^28^. Disease Ontology (DO) enrichment analysis was performed with an online tool Enrichr (https://maayanlab.cloud/Enrichr/). FDR < 0.05 was considered significant enrichment.

### Construction of PPI network

To characterize the crucial DEGs and clusters in skin scar, we used an online tool STRING (https://string-db.org/) to construct PPI networks with a minimum required interaction score of 0.4^29^. For further analysis, Cytoscape software^30^ was used with the download interaction information. Significant genes were determined by the cytoHubba plugin^31^ as hub genes.

### Expression patterns analysis of ferroptosis regulators

The list of genes involved in ferroptosis was retrieved from a review on this topic^32^. Then, expression patterns analysis of those ferroptosis regulators were conducted respectively in PsA-PsC and PsA-CL group. The results were shown in box plots, p < 0.05 was considered differentially expressed. The statistical difference of two groups was compared through the Wilcox test.

### Association analysis between differentially expressed ferroptosis regulators and hub genes

In order to discover an exclusive link in PsA, an association analysis between those differentially expressed ferroptosis regulators and hub genes was performed in PsA, PsC, and CL group respectively. For detail, all microarray data was downloaded from the GEO database. The raw data were downloaded as MINiML files. The R software pheatmap package was used to draw a multi-gene correlation. The expression correlation of two genes was analyzed with spearman. The abscissa and ordinate represent genes, different colors represent different correlation coefficients (red resents positive correlation whereas blue resents negative correlation), the darker the color, the stronger the relation. Asterisks (*) stand for significance levels, ** for p < 0.01, * for p < 0.05.

### Co-expression analysis of target genes in pan-cancer

ENCORI (https://starbase.sysu.edu.cn/)^33^ was utilized to do a co-expression analysis of target hub genes and major ferroptosis regulators that have exclusive linkages in PsA to further characterize the target genes. Target genes were identified based on the number of cancer species with |co-efficient R| ≥ 0.2 and P < 0.05.

### Expression analysis of CLEC2B in pan-cancer

We extracted the expression data of CLEC2B in various samples and then performed the log2 transformation of each expression value. We calculated the difference in expression between paired tumor and adjacent normal tissues as well as normal and tumor samples in indicated tumor types using R software (version 3.6.4) and analyzed difference significance using unpaired Wilcoxon Rank Sum Tests. Asterisks (*) stand for significance levels, ** for p < 0.01, * for p < 0.05. The results were visualized by ggplot^27^.

### Prognostic analysis of CLEC2B in pan-cancer

We obtained a high-quality prognostic dataset of TCGA from previous prognostic studies of TCGA published in Cell^34^. Furthermore, we performed log2(x + 0.001) transformation for each expression value, and finally we also excluded the cancers with less than 10 samples in a single cancer species. The connection between the CLEC2B expression and the prognosis of patients, including overall survival (OS) in multiple types of cancer was examined using forest plots and Kaplan–Meier curves. The hazard ratios (HRs) and 95% confidence intervals were calculated using univariate survival analysis.

### Correlation of the CLEC2B expression with immune cell infiltration and immune modulator genes

The gene expression profile of each tumor was extracted and mapped to GeneSymbol. Further, the infiltration score of each immune cell type in each tumor for each patient was re-evaluated based on gene expression, using the xCell^35^ and Timer^36^ methods of R package IOBR^37^. We calculated spearman's correlation coefficient between gene and immune cell infiltration scores in each tumor using the corr.test function of R package psych (https://CRAN.R-project.org/package=psych) to determine the significantly correlated immune infiltration scores. Additionally, the expression data of CLEC2B and 60 marker genes of two types of immune checkpoint pathway genes (Inhibitory(24), Stimulatory(36))^38^ in each sample was also explored, and the spearman correlation between CLEC2B and the marker genes of the five immune pathways was also calculated (*p < 0.05, **p < 0.01, ***p < 0.001).

## Results

### Data preprocessing

**Figure 2:**
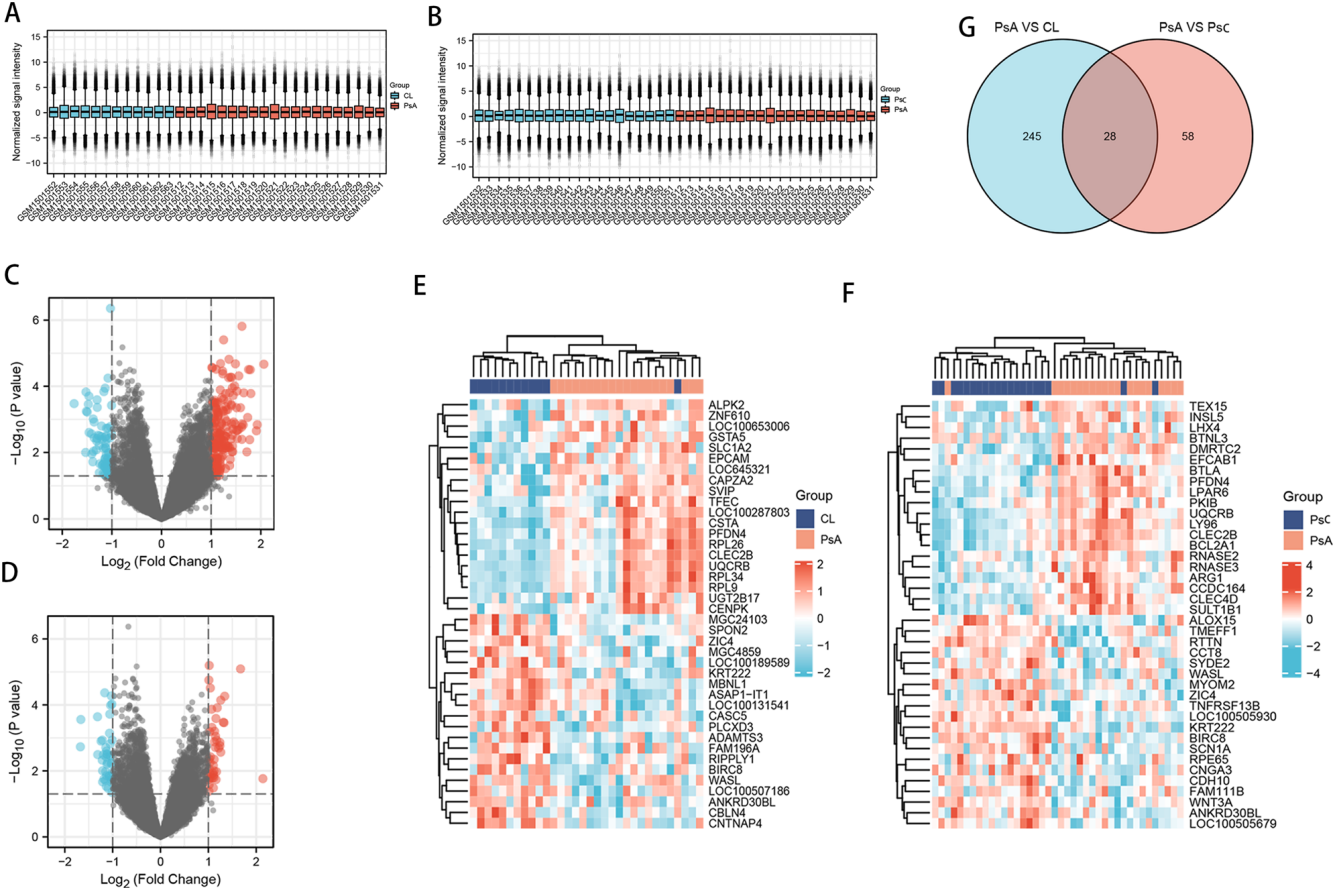
Identification of differentially expressed genes (DEGs). (**A**) Box plot after data standardization of PsA and CL group. (**B**) Box plot after data standardization of PsA and Ps group. Volcano plots of DEGS between the PsA and the CL (**C**) as well as PsA and PsC group (**D**). The red point in the plot represents the over-expressed mRNAs and the blue point indicates the down-expressed mRNAs with statistical significance. Heatmap of top 50 DEGs between PsA and CL group (**E**) and between the PsA and PsC group (**F**). (**G**) A Venn plot of DEGs overlapped between two groups.

After searching in Gene Expression Omnibus database with the inclusion criteria included: (1) patients with psoriatic arthritis or cutaneous psoriasis; (2) whole blood; 52 blood samples from GSE61281 were obtained (20 from patients with PsA, 20 from patients with PsC and 12 normal controls). We first performed background correction and data normalization after collecting the dataset, and the results are given in box plots (Fig. 2A,B).

### Identification of DEGs

After data preprocessing, DEGs were identified with the setting of cutoff at P < 0.05 and |log2 (FC)| ≥ 1. As shown in the volcano plots, totally 273 DEGs from PsA-CL group and 86 DEGS from PsA-PsC group were obtained (Fig. 2C,D). The top 50 DEGs from two sets are illustrated in heatmap plots (Fig. 2E,F). The overlapped DEGs between two sets are presented in a Venn diagram (Fig. 2G). A total of 28 overlapped DEGS were obtained and used for further analysis.

### Functional analysis and PPI network construction of DEGs

**Figure 3:**
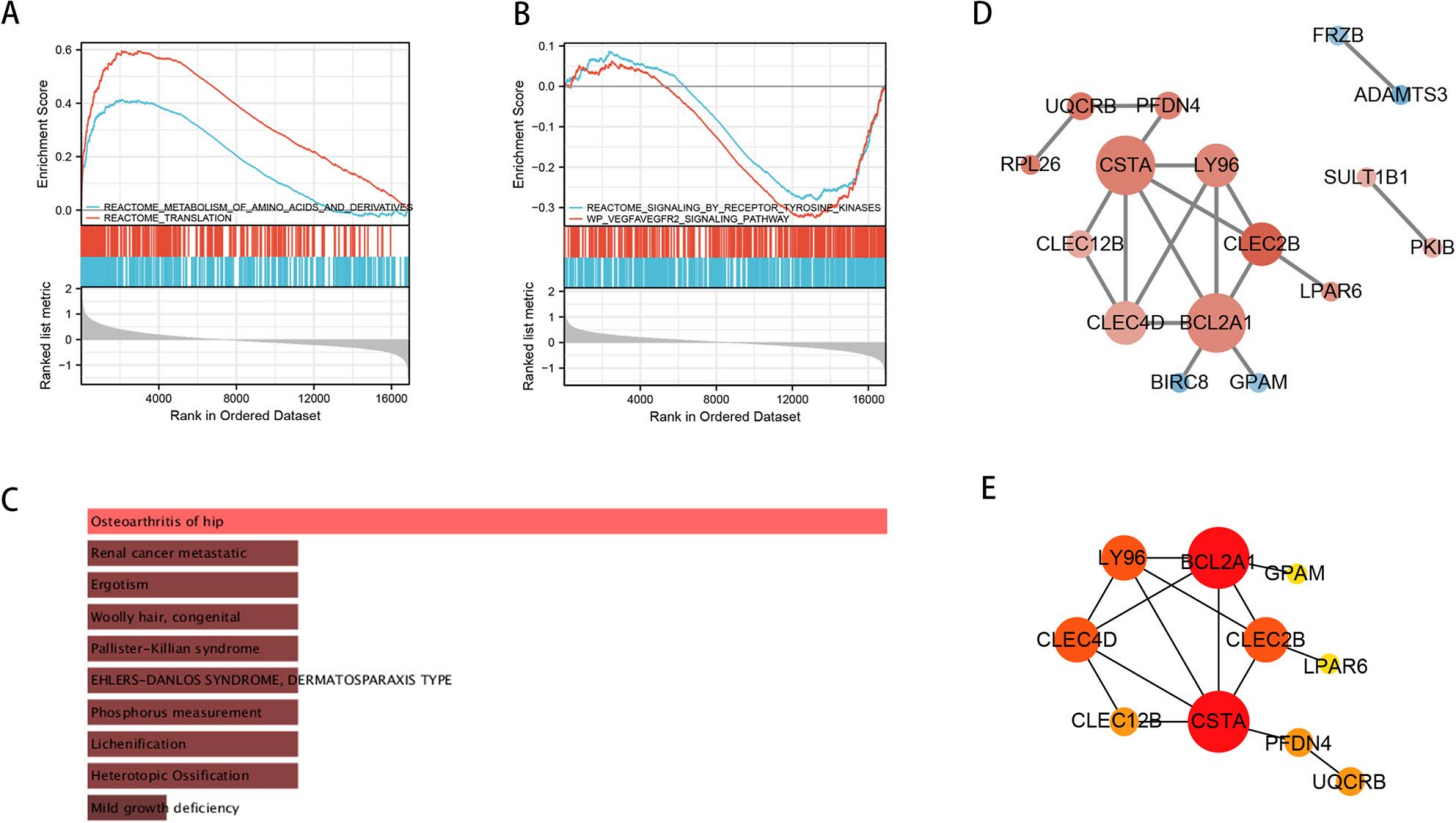
Enrichment analysis and PPI network construction of overlapped DEGs. (**A**) GSEA results of PsA and CL group; (**B**) GSEA results of PsA and PsC group; (**C**) Disease Ontology (DO) analysis of overlapped DEGs. (**D**) PPI network of overlapped DEGs. Red and blue circles represented up- and down-regulated genes, respectively. The edge color and width are proportional to the combined score analyzed by STRING. (**E**) The top 10 hub genes are determined by cytoHubba.

The result of GSEA analysis was shown in Fig. 3A (PsA-CL) and Fig. 3B (PsA-PsC). As for DO enrichment analysis, overlapped DEGs mainly involved in Osteoarthritis of hip (Fig. 3C). Further, to study the interaction of overlapped DEGs, the PPI network was built using STRING and then visualized in Cytoscape (Fig. 3D). Subsequently, cytoHubba plugin was used to characterize the hub genes. The top 10 genes based on the filtering algorithm (degree) were shown in Fig. 3E.

### Expression profile of selected ferroptosis regulators

**Figure 4:**
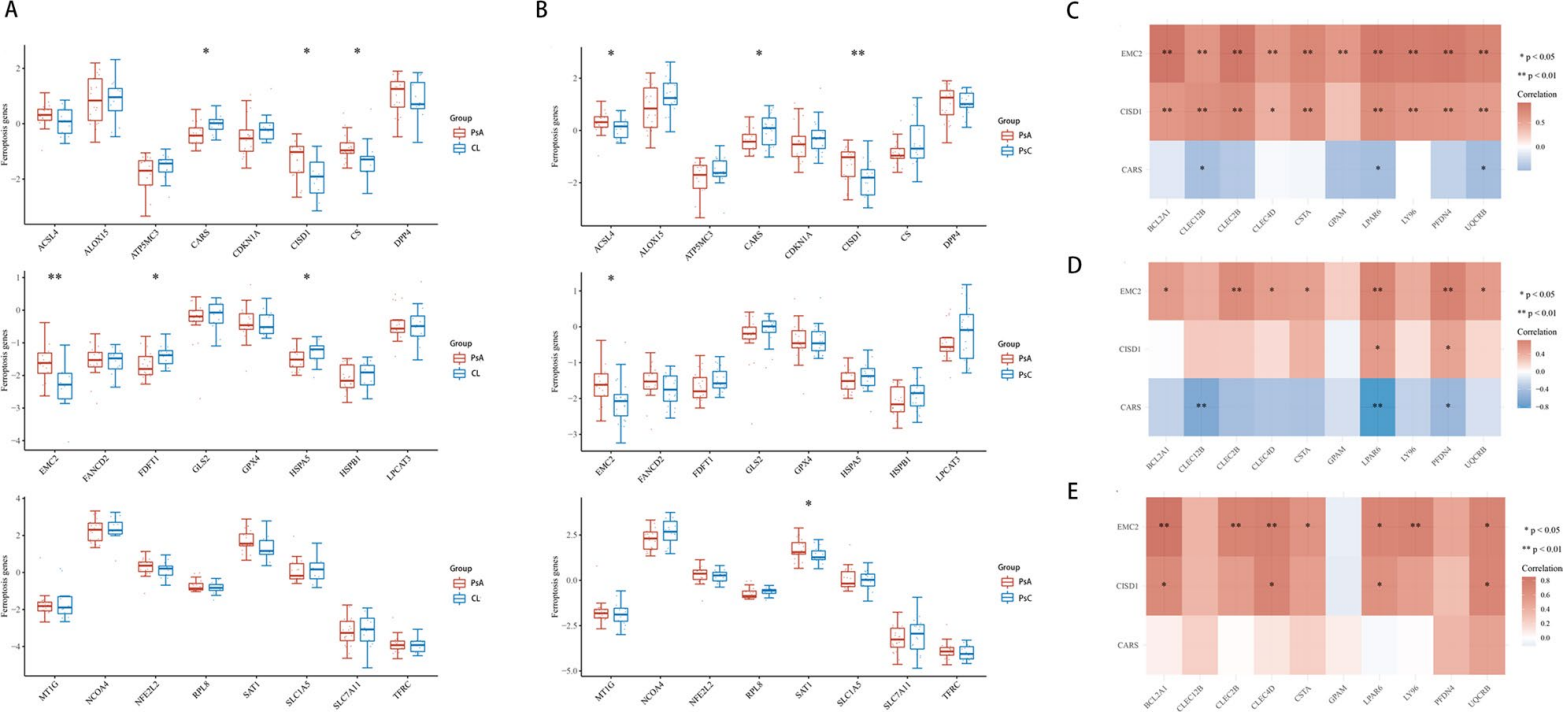
Ferroptosis-related analysis. The expression distribution of Ferroptosis-related mRNA in PsA and CL group (**A**), in PsA and PsC group (**B**). The horizontal axis represents different mRNA, the vertical axis represents the mRNA expression distribution, and the upper left corner represents the significance p-value test method. (**C**–**E**) Correlation between hub genes and differentially expressed ferroptosis regulators in PsA group (**C**), PsC group (**D**) and normal control (**E**). The horizontal axis represents 10 hub genes, the vertical axis represents 3 ferroptosis regulators. Red represents positive correlation, blue represents negative correlation, and the darker the color represents the two stronger correlation. Asterisks represent levels of significance (*p < 0.05, **p < 0.01).

Differences in the expression levels of 24 selected ferroptosis regulators were compared between PsA and PsC as well as PsA and CL groups, A difference was deemed significant with P < 0.05 (*p < 0.05, **p < 0.01). Compared with PsC, the expression levels of ACSL4, CISD1, EMC2 and SAT1 were increased in PsA group, but the level of CARS was decreased (Fig. 4B). While compared with CL, the expression level of CISD1, CS, EMC2 were significantly increased (Fig. 4A), while CARS, FDFT1 and HSPA5 were found decreased-expressed. Based on these findings, CISD1, EMC2, and CARS were identified as key ferroptosis regulators that were linked to PsA.

### Association analysis of key ferroptosis regulators and hub genes

We then tried to investigate the correlation between 10 hub genes and 3 key ferroptosis regulators with spearman (*p < 0.05, **p < 0.01, ***p < 0.001). As shown in Fig. 4C, in PsA group, among the 3 key ferroptosis regulators, EMC2 showed significantly positive association with all 10 hub genes, CISD1 also showed positive association with 9 hub genes. CARS showed negative association with 3 hub genes (CLEC12B, LPAR6 and UQCRB). As compared to the multiple-gene association results of the PsC group (Fig. 4D) and the CL group (Fig. 4E), CISD1 revealed a distinct positive connection with CLEC12B, CLEC2B, and CSTA exclusively in the PsA group.

### Pan-cancer co-expression analysis for the ferroptosis regulators-hub genes interactions across 32 types of cancers

**Figure 5:**
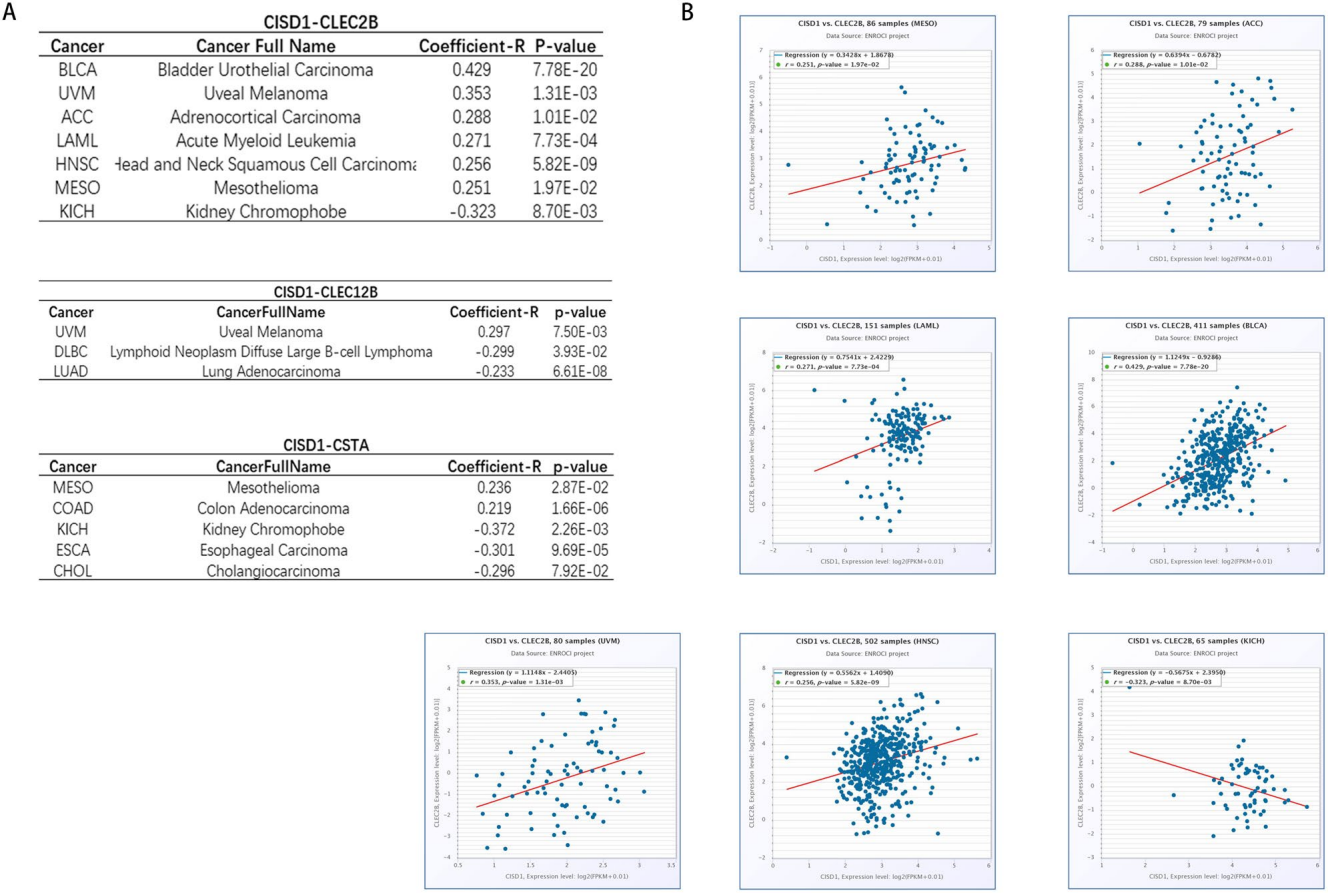
Co-expression analysis of target genes in pan-cancer. (**A**) Cancers with |co-efficient R| ≥ 0.2 of CISD1-CLEC2B/CLEC12B/CSTA co-expression analysis; (**B**) Scatterplots of cancers with |co-efficient R| ≥ 0.2 of CISD1-CLEC2B co-expression analysis.

After investigating the pan-cancer section of ENCORI, an online tool, CISD1 was discovered to be highly linked to CLEC2B, CLEC12B and CSTA in a variety of cancer types. We also used |Coefficient-R| ≥ 0.2 as well as p < 0.05 to retrieve the cancer kinds and showed the findings in Fig. 5A. Figure 5B showed the detailed results of each cancer type's co-expression study of CLEC2B. Based on this, we infer that CLEC2B may be the most likely gene associated with ferroptosis in psoriatic arthritis, which is linked to a high risk of cancer.

### Functional correlation analysis of CLEC2B and CISD1

**Figure 6:**
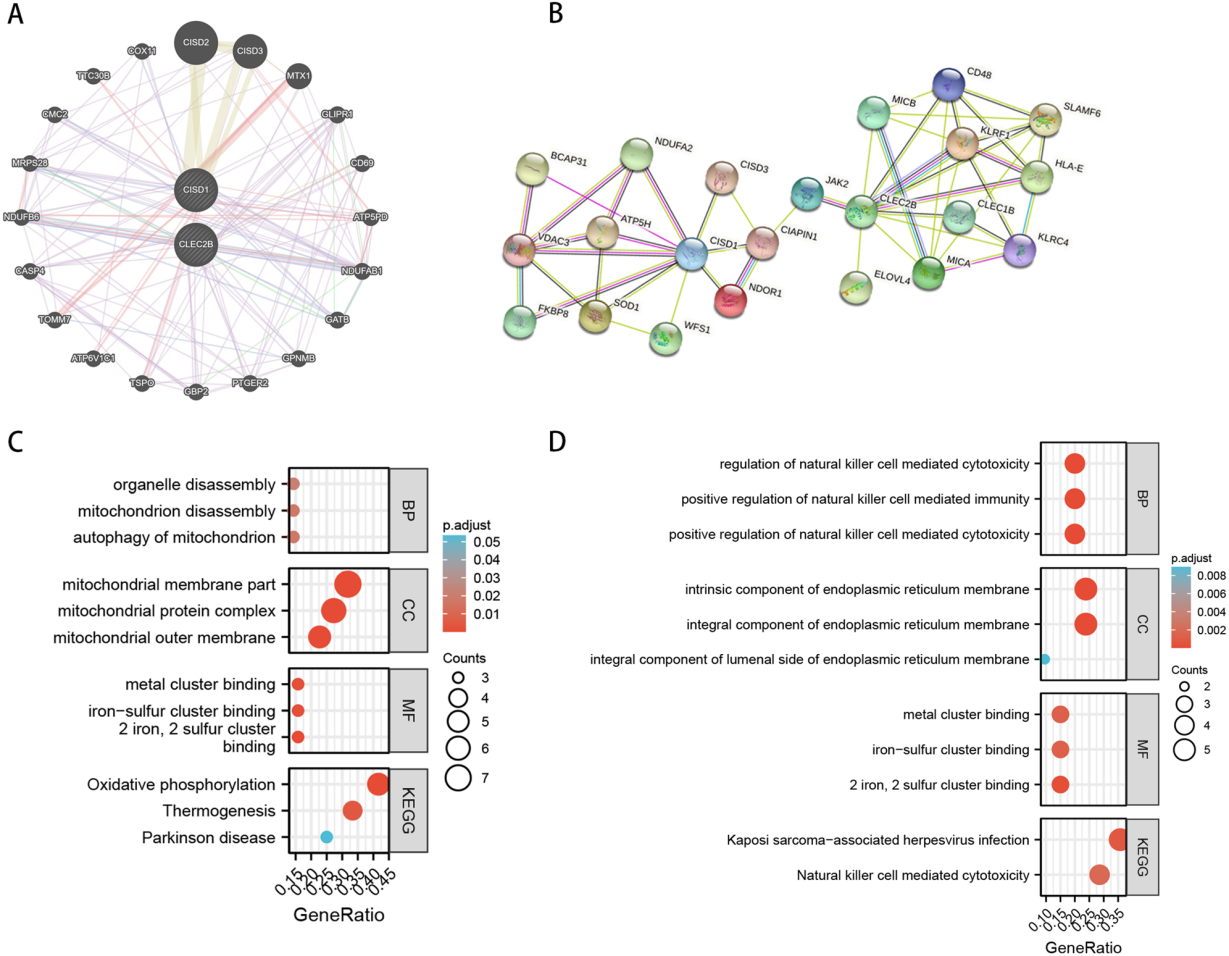
Functional correlation analysis of CLEC2B and CISD1. (**A**) PPI network from GeneMANIA after searching with CLEC2B and CISD1. (**B**) PPI network form STRING after searching with CLEC2B and CISD1. GO/KEGG enrichment analysis of gene lists from GeneMANIA (**C**) and STRING (**D**). In the enrichment result, FDR < 0.05 is considered to be enriched to a meaningful pathway. BP: The biological process; CC: cellular component; MF: molecular function.

GeneMANIA^39^ and STRING^29^ were used to obtain correlated gene sets of CLEC2B and CISD1 (Fig. 6A,B). Then the two gene lists were used for enrichment analysis, respectively. Enriched GO terms were divided into three categories: molecular function (MF), biological process (BP), and cellular component (CC). The gene list from GeneMANIA was mostly enriched in 'iron-sulfur cluster binding' in the MF group, and 'Oxidative phosphorylation' in the KEGG group, as shown in Fig. 6C. In terms of the STRING gene list, the most prominent results were 'positive regulation of natural killer cell mediated cytotoxicity, immunity' in the BP group, 'iron-sulfur cluster binding' in the MF group, and 'Natural killer cell mediated cytotoxicity' in the KEGG group (Fig. 6D).

### CLEC2B expression analysis in pan-cancer

**Figure 7:**
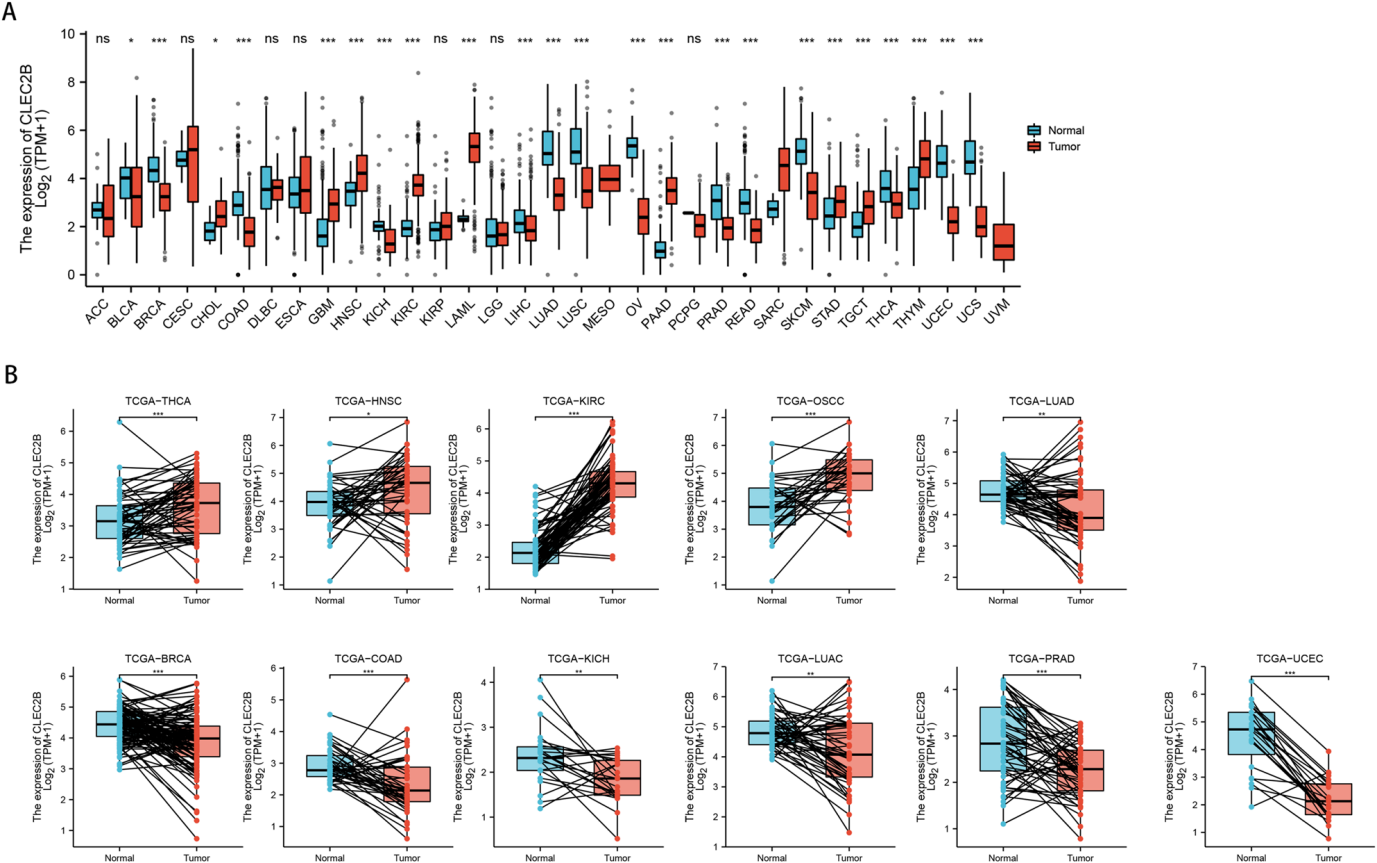
CLEC2B expression analysis in pan-cancer. (**A**) Unpaired expression differences of CLEC2B in pan-cancer from TCGA + GTEx; (**B**) expression differences of CLEC2B in paired tumor and normal tissues in TCGA pan-cancer.

We calculated the difference in expression between normal and tumor samples in each tumor using R software (version 3.6.4) and difference significance analysis using unpaired Wilcoxon Rank Sum and Signed Rank Tests. As shown in Fig. 7A, we observed significant upregulations in 10 tumors, such as CHOL, GBM, HNSC, KIRC, LAML, PAAD, SARC, STAD, TGCT, and THYM; and significant deregulation in 14 tumors including BLCA, BRCA, COAD, KICH, LIHC, LUAD, LUSC, OV, PRAD, READ, SKCM, THCA, UCEC and USC. For paired tumor and normal tissues in TCGA pan-cancer (Fig. 7B), CLEC2B was expressed at high levels in 4 tumors and low levels in 7 tumors.

### Prognostic significance of CLEC2B in pan-cancer

**Figure 8:**
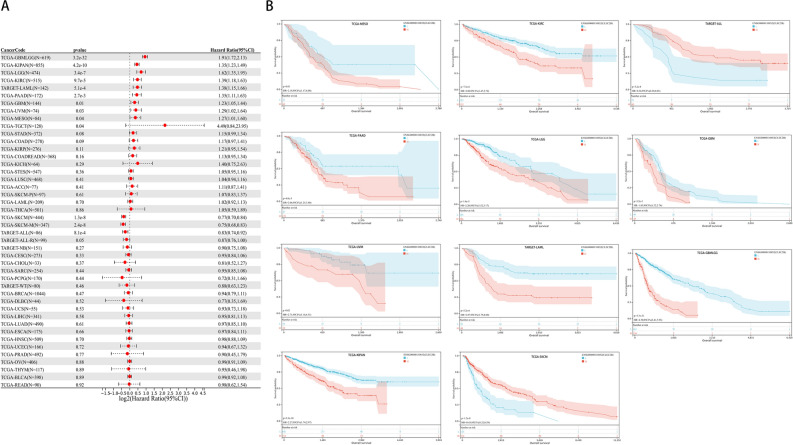
Association between the CLEC2B expression and OS in cancer patients. (**A**) A forest plot of hazard ratios of CLEC2B in 32 types of tumors. (**B**) Kaplan–Meier survival curves of OS for patients stratified by the different expressions of CLEC2B.

We used the coxph function of the R package to build a Cox proportional hazards regression model to analyze the relationship between CLEC2B expression and prognosis in each tumor, and statistical tests were performed using Logrank test to obtain prognostic significance. We finally observed that upregulated CLEC2B expression was remarkably associated with poor OS in 10 tumor types (GBMLGG, KIPAN, LGG, KIRC, LAML, PAAD, GBM, UVM, MESO, TGCT) and downregulated expression was correlated with poor OS in 2 tumor type (SKCM and ALL). The results of Kaplan–Meier analysis were consistent with the above findings (Fig. 8).

### Analysis of the CLEC2B expression and immunological correlation in pan-cancer

**Figure 9:**
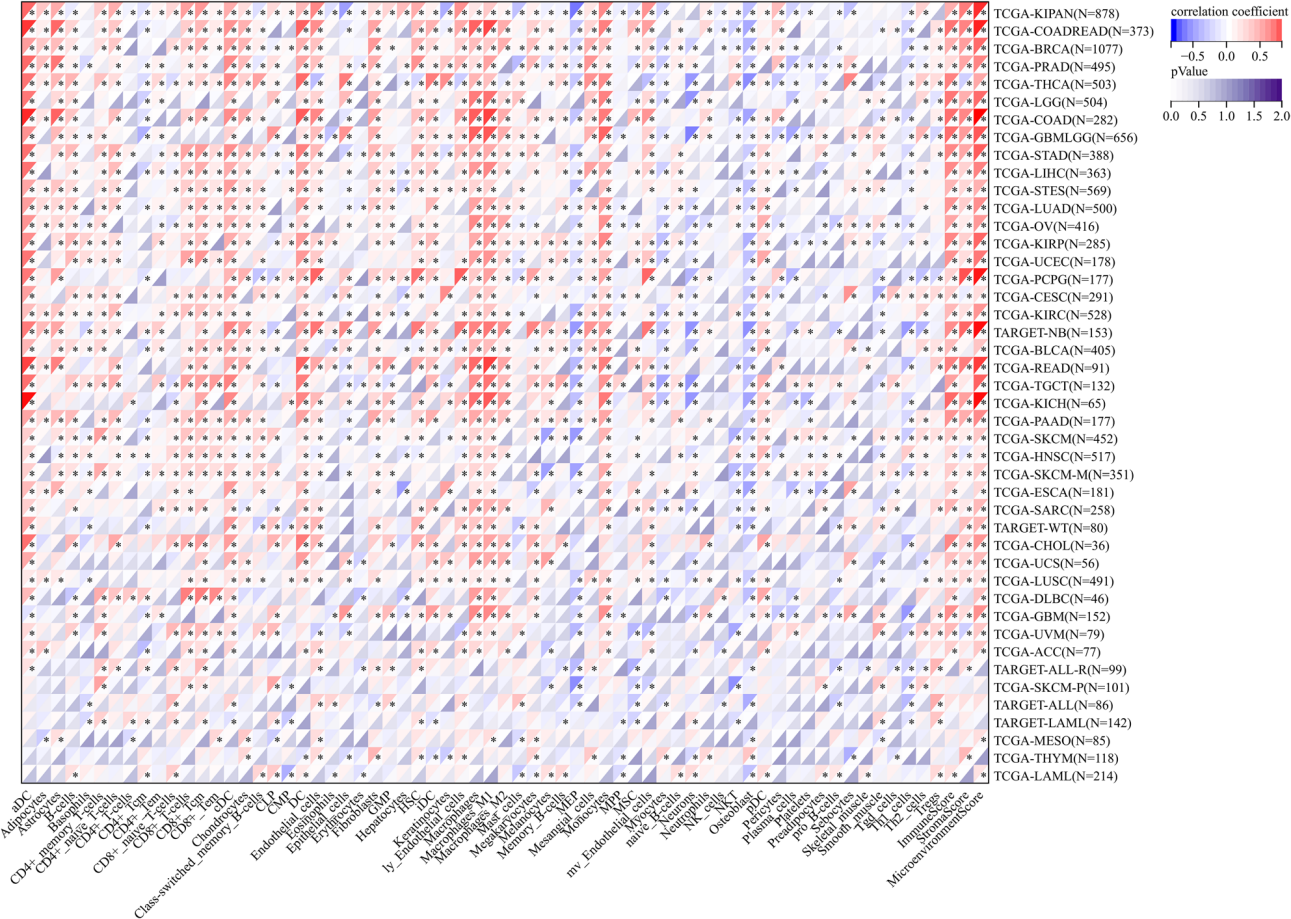
Association of CLEC2B expression and immune cell infiltration using XCELL method.

**Figure 10:**
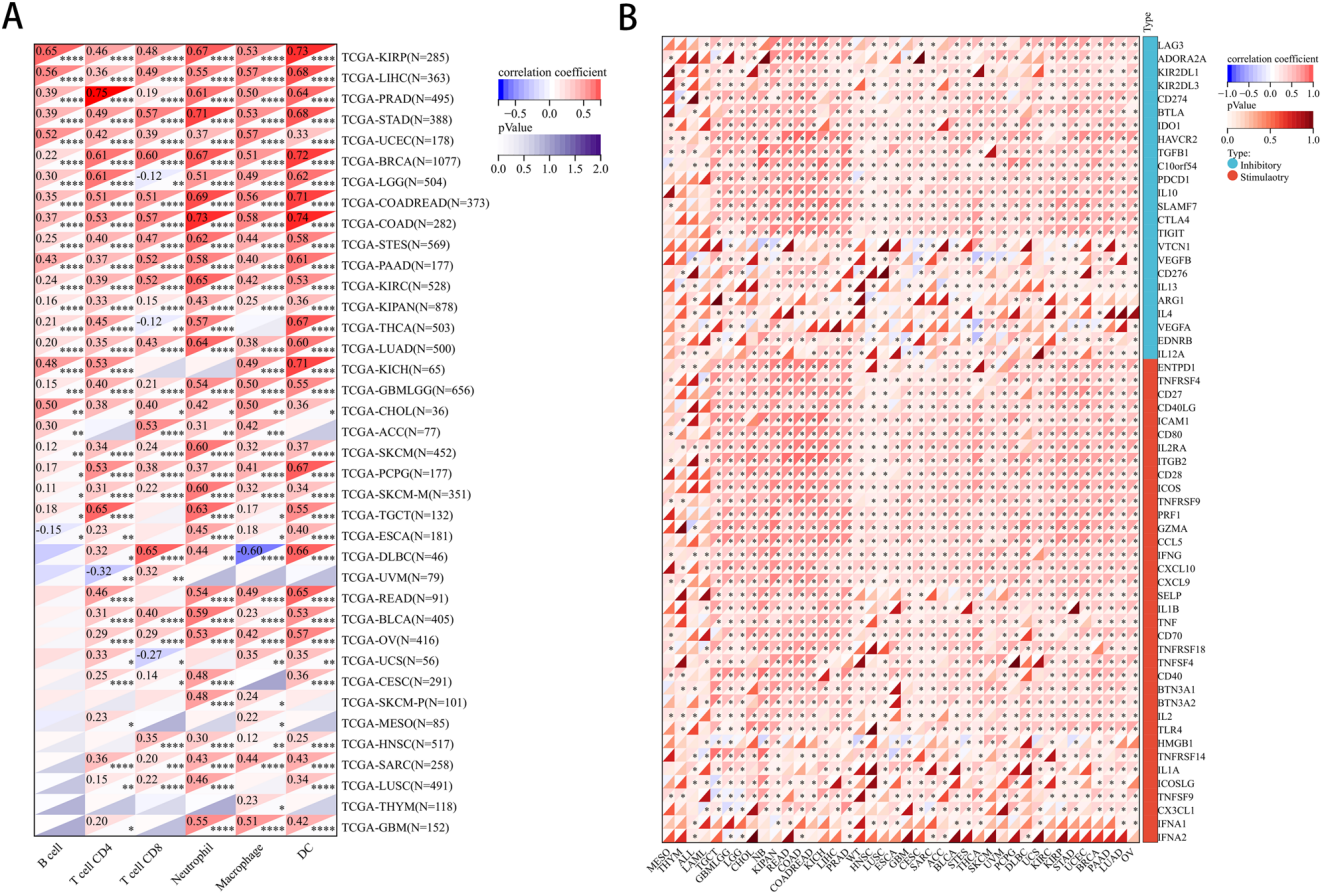
(**A**) Association of CLEC2B expression and immune cell infiltration using TIMER method. (**B**) The correlation between CLEC2B and immunoregulation-related genes.

Among 38 subtypes of immune cells, we found that the CLEC2B expression significantly correlated with most subtypes in different tumor types (Fig. 9). As shown in Fig. 10A, the expression of CLEC2B was significantly associated with the abundance of infiltrating immune cells using TIMER method: B cells in 24 types of cancer, CD4+ T cells in 29 types of cancer, CD8+ T cells in 30 types of cancer, neutrophils in 34 types of cancer, macrophages in 33 types of cancer, and DCs in 33 types of cancer. We further used the deconvo_xCell method of the R package IOBR to calculate the infiltration scores of various types of immune cells. Immunosurveillance influences the prognosis of cancer patients, and tumors evade immune responses by taking advantage of immune checkpoints. Our findings revealed that CLEC2B was correlated with most immunoinhibitors and immunostimulators in multiple cancer types (Fig. 10B), suggesting that CLEC2B could collectively influence the prognosis of multiple cancers through immunosurveillance.

## Discussion

The risk of cancer developing and incidence in people with psoriasis disease has raised some concern^40,41^. Contrary to the established nature of the associations between these comorbidities including depression and cardiovascular disease, the underlying mechanism of high cancer risk and psoriasis disease appear to be much less well-established^42^. Even less is known about whether psoriatic arthritis raises the risk of cancer in psoriasis sufferers. Given the importance of ferroptosis in cancer development as well as inflammatory response in psoriasis^43^, we attempted to employ bioinformatics analysis to investigate the association between ferroptosis and psoriasis disease, specifically psoriatic arthritis.

In this investigation, a total of 10 hub genes (Fig. 3E) and 3 differentially expressed ferroptosis regulators (CISD1, EMC2, CARS) were obtained through a series of analysis. Going to follow that, association analysis was performed for the aforementioned genes. The results revealed that CISD1 was associated with CLEC12B, CLEC2B and CSTA only in PsA. We therefore postulate that the four genes could serve as distinctive indicators that distinguish psoriatic arthritis from cutaneous psoriasis. Among them, CISD1 was also found to be closely linked to CLEC12B, CLEC2B and CSTA in a variety of cancers, with CLEC2B being involved in the largest number of cancer types. CLEC2B, a member of the C-type lectin domain family 2 (CLEC-2) that was formerly known as activation-induced C-type lectin (AICL), is a protein encoded by the natural killer (NK) gene complex proximal CD69 that has a high level of expression during lymphocyte activation^44^. Our results similarly demonstrate the relationship between CLEC2B and natural killer cells. Additionally, we discovered that CLEC2B collaborated with the ferroptosis regulator CISD1 to connect iron-sulfer cluster binding and oxidative phosphorylation, further demonstrating the strong connection between CLEC2B and ferroptosis.

Based on aforementioned results, we further explored the function of CELC2B in pan-cancer. In previous studies, CLEC2B has been identified as a marker for a variety of cancers, including clear cell renal cell carcinoma, melanoma, and pancreatic adenocarcinoma^45,46^. Similar to the findings of prior studies, this study found vital functions for CLEC2B in numerous cancers, including changes in expression, correlation with prognosis, infiltration of multiple immune cells and even immune checkpoints in multiple cancer types. Given that, CLEC2B was considered as a key gene which bridges the gap between the development of psoriatic arthritis and cancer through ferroptosis. However, the link between CLEC2B and ferroptosis regulator CISD1 in various malignancies varied in strength and type, which implies that even the same two genes function differently under various regulatory systems in malignant tumors, demonstrating the complexity and unknowable nature of cancer etiology.

This is the first time that CLEC2B has been found to be associated with the development of psoriatic arthritis through natural killer cells. More importantly, as a key gene exclusively linked to ferroptosis regulators in psoriatic arthritis, CLEC2B is differentially expressed in a variety of cancers and is closely associated with immune cell infiltration as well as immune checkpoints. This study may provide new research insights into the high cancer risk in patients with psoriatic psoriasis.

## Data Availability

The datasets generated and analysed during the current study are available in the Gene Expression Omnibus database (http://www.ncbi.nlm.nih.gov/geo).
